# Evaluation of a Novel WeChat Applet for Image-Based Dietary Assessment among Pregnant Women in China

**DOI:** 10.3390/nu13093158

**Published:** 2021-09-10

**Authors:** Ye Ding, Xiaolong Lu, Zhencheng Xie, Tingting Jiang, Chenglin Song, Zhixu Wang

**Affiliations:** Department of Maternal, Child and Adolescent Health, School of Public Health, Nanjing Medical University, Nanjing 211166, China; dingye@njmu.edu.cn (Y.D.); lu123xiaolong@163.com (X.L.); zhenchengxie@njmu.edu.cn (Z.X.); ttj96go@126.com (T.J.); scl_song@163.com (C.S.)

**Keywords:** Chinese pregnant women, image-based dietary assessment, WeChat Applet, relative validity, dietary recall

## Abstract

As an important part of antenatal care for pregnant women in China, dietary assessment plays a positive role in maternal and fetal health. Shortcomings in the associated methodologies require improvement. Our purpose was to develop a novel WeChat Applet for image-based dietary assessment (WAIDA) and evaluate its relative validity among pregnant women in China. Data on 251 lunch meals of pregnant women in their second trimester were analyzed. The differences in food weight, energy, and nutrient estimates by the dietary recall or WAIDA method with the weighing method were compared using paired t-tests. Pearson correlation coefficients were used to analyze the correlation between food weight, energy, and nutrient intake obtained from the recall or WAIDA method and those obtained from the weighing method. The Bland–Altman analysis was used to examine the agreement between the recall or WAIDA method and the weighing method for energy and nutrients. Compared with the weighing method, the variation range of food weight, energy and nutrients estimated by the WAIDA method was smaller and more stable than that estimated by the recall method. Compared with the recall method, the correlations suggested a better relationship between the energy and nutrient intakes from the weighing method and those estimated by the WAIDA method (0.752–0.970 vs. 0.480–0.887), which were similar to those of food weight (0.332–0.973 vs. −0.019–0.794). The Bland-Altman analysis showed that the mean differences of the energy and nutrients estimated from the recall method were further away from zero relative to the weighing method compared to the WAIDA method and with numerically wider 95% confidence intervals. The spans between the upper and lower 95% limit of agreement (LOAs) of the energy and nutrients obtained by the WAIDA method were narrower than those obtained by the recall method, and the majority of the data points obtained by the WAIDA method lay between the LOAs, closer to the middle horizontal line. Compared with the recall method, the WAIDA method is consistent with the weighing method, close to the real value of dietary data, and expected to be suitable for dietary assessment in antenatal care.

## 1. Introduction

Diet quality plays an important role in the health of pregnant women and can also influence fetal development, birth outcomes, and both the early health status and lifelong disease risk in offspring [1,2]. Dietary assessment is an important part of antenatal care for pregnant women in China, which can provide valuable insights into the occurrence of disease and subsequent approaches for prevention and intervention. The weighing food record method is widely accepted as the best method of dietary assessment, and is used as the golden standard to evaluate the relative validity of other dietary assessment methods. However, this method is quite expensive and has a high respondent burden (including time and manpower), which often leads to low participation rates and high exit rates [3,4,5]. Therefore, it is generally only used for pregnant women with nutrition-related diseases, such as gestational diabetes mellitus, and is not suitable for healthy pregnant women. The 24-hour dietary recall (24 HR) method is the most common method for prenatal care of pregnant women in China, and is simple and easy to use. However, the 24 HR relies on the respondents’ self-report; the respondents (especially young people) are limited in their ability to identify food types and estimate the amount of food accurately; this cannot avoid the recall bias [6]. Therefore, based on current technology, much effort has been put into the development of new dietary assessment methods as a feasible solution to solve the current methodological defects. In recent years, the use of images as the main record of dietary intake has shown several advantages [7,8,9]. Images may increase objectivity, reduce the burden on respondents related to the collection of dietary intake information, and help to avoid retrospective bias. These advantages make the image-based dietary assessment particularly suitable for antenatal care in pregnant women, for which time and personnel conditions are very limited when implemented due to overpopulation and limited medical resources in China.

Recently, researchers have been particularly interested in using smartphones to assist in dietary assessment [10,11]. Smartphones have many advantageous technological features, including wireless communication, built-in cameras, portable designs, and connectivity to external devices via Bluetooth, making them a convenient and suitable platform for image-based dietary assessment. In China, smartphone ownership has grown exponentially over the past decade. According to a statistical report on China’s Internet development, by December 2020, the number of Internet users was 989 million, of which 986 million (99.7%) used smartphones to access the Internet [12]. WeChat is a type of social networking software that provides instant messaging services on smart terminals, and is very popular among young groups in China. In 2020, the number of monthly active users of WeChat exceeded 1.1 billion, making it the most common smartphone application in China. It is no longer a simple social platform, but has penetrated into all aspects of people’s lives. With the continuous development of WeChat development tools, a new development environment and platform was built for the WeChat Applet used by 400 million Chinese users every day [13,14,15,16]. The WeChat Applet, also known as the Mini Program, has great advantages. The use steps are simplified, and they can be opened directly without downloading the application package. Interestingly, there is an independent storage space between the different applets. If you no longer use the applet, you just need to close the page, without needing to uninstall the program or clear the cache, which is convenient for users, and can also reduce the memory footprint. In addition, this technology can bring about a rapid transfer of digital data between pregnant women and doctors, and reduce their burden related to the collection of dietary intake information through real-time communication [17,18]. All these provide convenience for the development and use of image-based dietary assessment method among pregnant women, a young population.

At present, there has been no report on the WeChat Applet for image-based dietary assessment, but the smartphone-based image dietary record has been used, and the obtained dietary data have been proved to be valid and credible. However, dietary habits vary greatly in populations with different regional, ethnic, dietary, or cultural backgrounds. Compared with Chinese food, the food ingredients of Western countries are relatively simple and so are the image-based dietary assessments they use. For example, the food types considered by researchers when verifying the food data collected from images are usually scattered and unmixed, such as chicken nuggets, sandwiches, or biscuits [19], and the validity of the method is generally compared with the weighing method using images taken only at the front from a 45° angle. There is a great variety of Chinese food, and the processing and cooking methods are complex and diverse. Most dishes are a mixture of various ingredients, which makes dietary evaluation difficult to estimate [20,21]. Therefore, the assessments mentioned above are not applicable to pregnant women in China. We emphasize that our goal was to develop a novel WeChat Applet for image-based dietary assessment (WAIDA) among pregnant women in China. Moreover, we assessed the relative validity of the WAIDA by analyzing the difference between the WAIDA and recall method when compared with the actual food weight.

## 2. Materials and Methods

### 2.1. Overview of the WAIDA

**Figure 1:**
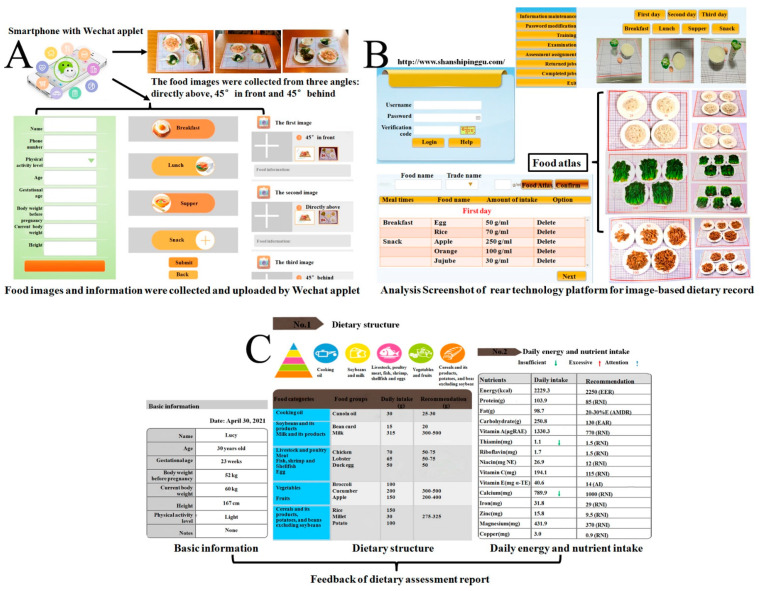
Overview of the WeChat Applet for Image-based Dietary Assessment (WAIDA). To collect meal information, images are taken from three angles using the built-in smartphone camera and uploaded through the WeChat Applet to a web server (**A**). Analysis consists of the dietary assessor identifying and quantifying the food in each image. The food atlas is used to assist in food quantification (**B**). Dietary data is entered directly into the database to obtain the intake of each food category and estimate the energy and nutrient intake. The basic information of pregnant women, the rationality of dietary structure, and the daily intake of energy and nutrients are then fed back (**C**).

The WAIDA includes two data input systems including a web and mobile phone, a back-end data processing system, a report output system, a nutrition database, and a food atlas. (Figure 1). The specific operation is as follows: users download the WeChat software on their smartphones, scan the QR code or search “dietary assessment” to enter the application interface, and complete the basic information in the initial interface, such as the name, age, pregnancy week, height, weight, etc. Users then enter the upload interface and upload images of their breakfast, lunch, supper, and snack, according to the prompt information of the interface. Each meal needs to be uploaded in three images from three angles before and after the meal, and user must provide the name of the food, the composition of the food mixture, etc. After uploading the images, users need to verify the dietary information again and fill in the missing information, such as the use of edible oil, dietary supplements, and snacks. Finally, all data are uploaded to the web server. The server contains a subsystem management, dietary assessment, food atlas, and food composition database. After the dietary information enters the server, the subsystem administrator manages the data and assigns tasks to the dietary assessor. According to the food atlas used to assist in food quantification, the dietary assessor identifies and estimates each type of food in each image by themselves. After collecting the dietary information for one day, the assessor summarizes the food categories and compares them with the dietary recommendations of the 2016 Chinese Balanced Dietary Pagoda [22] for pregnant women to evaluate the dietary structure. At the same time, the data are indexed by the food composition database, and the daily energy and nutrient intakes are calculated and compared with the 2013 Chinese Dietary Reference Intakes [23] for evaluation. Finally, the results are fed back to the users and summarized in the database. The food atlas was specially developed by our research group for the WAIDA method to assess the portion sizes. It contains 207 images of 69 food types. Each type of food is presented in three images from three different angles (directly above, at a 45° angle in front, and at a 45° angle behind), and each photo shows four to six different food portions. The establishment of a food composition database is based on the Chinese Food Composition Table (6th edition) [24].

### 2.2. Validation of the WAIDA

#### 2.2.1. Participants

An observational study was conducted to validate the WAIDA. From November 2020 to April 2021, pregnant women in their second trimester (13–27 weeks) were invited to participate in the study at the maternity clinics of Danyang People’s Hospital, Jiangsu Province, China. Healthy women with singleton pregnancies, no clinical diagnosis of infectious disease, a history of metabolic disorders (obesity, hypertension, diabetes, etc.), a history of pregnancy complications (hyperemesis gravidarum, gestational hypertension, gestational diabetes, etc.), and malnutrition disorders (osteoporosis, anemia, iodine deficiency, goiter, etc.) were eligible for inclusion in the present study. Pregnant women who could not report their dietary intake due to a limited cognitive capacity or inability to use a smartphone were excluded. All subjects gave their informed consent for inclusion before they participated in the study. The study was conducted in accordance with the Declaration of Helsinki, and the protocol was approved by the Ethics Committee of Nanjing Medical University (2020-574).

#### 2.2.2. Study Design

**Figure 2:**
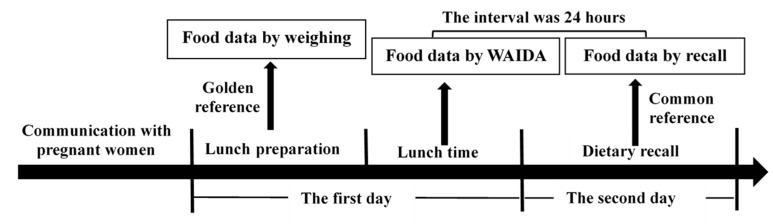
The design of the study investigating 251 pregnant women in Danyang, China.

All the researchers received at least 2 days of technical skill training before conducting the study, and only those who passed the examination could participate in this study. The study design is illustrated in Figure 2. The day before the observational study began, the researchers explained the process of the study and the matters requiring their cooperation with pregnant women. Pregnant women were told that they would be fed a lunch meal on the first day and then to return the second day to respond to some questions about food they ate. On the first day, the researchers prepared a lunch to obtain the weighing data of the food. When pregnant women had lunch, the WAIDA was used to obtain food data. On the second day, the researchers conducted a dietary recall to obtain the recall data. The interval between the WAIDA and dietary recall was 24 h. All researchers were divided into four independent working groups: lunch preparation, lunch service, dietary recall, and dietary assessment. The personnel and information in each group were completely isolated.

#### 2.2.3. Lunch Preparation and Food Weighing

A meal was cooked based on the lunch recipe of the day in the hospital canteen. On the same day, pregnant women were provided with the same type of food, but the amount of food was different for each person. The amount of food supplied and consumed refers to the weight of all types of food that can be 100% consumed in its classical state. For example, fruits and vegetables are 100% edible weight in their fresh state, and cereals and their products are 100% edible weight in their dry state. For preparation lunch, each food was cooked separately and then mixed into dishes. Before and after cooking, the raw and cooked weights of each food type were accurately weighed, and the corresponding raw/cooked weight ratio was calculated. When lunch was served, the weight of each food type was recorded before being mixed into a clean covered lunch box marked with the number of the participant. Finally, the food intake of each pregnant woman was calculated according to the cooked weight of each type of food before and after meals and the raw/cooked weight ratio. If there were still some inedible parts, such as bones after cooking, the 100% edible weight of each kind of food was calculated according to the proportion of edible parts provided by the Chinese Food Composition Tables (6th edition) [24].

#### 2.2.4. The WAIDA Method

Each participant was provided a two-dimensional background paper (scale with 1 cm × 1 cm) and flat plates. Our researchers explained to them how to record food images using the built-in smartphones camera, and upload images using the WeChat Applet, including oral and written descriptions of the method and specific operation video, in order to promote their cooperation and ensure a high quality of data obtained. Before lunch, the food was placed on a flat plate. The plate was then placed in the red area of the background paper. Next, the food was photographed from three angles: directly above, at a 45° angle in front and at a 45° angle behind. Pregnant women were instructed to display the entire red area of the background in all images. After lunch, the same method was used to photograph the unfinished food. Finally, food images and food descriptions (such as food name, ingredients of food mixture, etc.) were uploaded to the server through the WeChat Applet. Lunch records of pregnant women were checked in time. For missing or unclear images, pregnant women were asked to take the food images again to ensure the relative validity of the results.

#### 2.2.5. The Recall Method

As a part of the study, the lunch recalls were collected on the second day. Well-trained researchers interviewed pregnant women face-to-face. In addition to the metric cups and spoons, food models were provided to aid in the estimation of food and beverage portion sizes. The participants’ responses were recorded on a dietary recall form. After the interview, the researchers checked for any unclear information that the pregnant women had returned and resolved any issue [25].

### 2.3. Statistical Analysis

All data were calculated and analyzed using SPSS, version 20.0 (IBM Corp., Armonk, NY, USA). Statistical significance was set at *P* < 0.05.

According to the Chinese Balanced Dietary Pagoda for pregnant women and Chinese dietary habits, the food was divided into different groups, and the food weight estimated by the recall method or WAIDA method was compared with the actual food weight. According to the nutritional needs of women during pregnancy, energy and some important nutrients were selected. The proportion of energy and nutrient content of each food was established from the Chinese Food Composition Table, and energy and nutrient intakes derived from the recall or WAIDA method were compared with those derived from the weighing method. The relative difference (d) was calculated as d = data derived from the recall or WAIDA method – data derived from the weighing method. Meanwhile, the absolute difference (D) was calculated as D = |data derived from the recall or WAIDA method − data derived from the weighing method|. Subsequently, the percentage of d was calculated as d% = [(data derived from the recall or WAIDA method − data derived from the weighing method)/data derived from the weighing method] × 100. Finally, the percentage of D was calculated as D% = (|data derived from the recall or WAIDA method − data derived from the weighing method |/data derived from the weighing method) × 100. All differences were expressed as the mean ± standard deviation (SD). For each food group, energy, and each nutrient, the differences in food weight, energy, and nutrient estimates between the recall method and WAIDA method were compared using paired t-tests. Pearson correlation coefficients were used to analyze the correlation between food weight, energy, and nutrient intake obtained from the recall or WAIDA method and those obtained from the weighing method. Furthermore, on the premise that the distribution of differences between the recall or WAIDA method and weighing method was normal, the Bland–Altman analysis was used to examine the agreement between the recall or WAIDA method and the weighing method for energy and nutrients. The 95% confidence interval of the difference was calculated to observe the dispersion trend of the difference, and the 95% limit of agreement (LOA) was also investigated.

## 3. Results

### 3.1. Comparison and Correlation Analysis between Food Weights Estimated by the Recall or WAIDA Method with the Actual Food Weight

Because the participants’ recipes are not consistent and the food varieties are different, in the data processing, the food data of each participant are classified and summarized based on the Chinese Balanced Dietary Pagoda for pregnant women. The comparison was performed for four major food categories: cereals and potatoes (*n* = 269), fish, shrimp, shellfish, eggs, livestock meat, and poultry (*n* = 528), fruits (*n* = 286), and vegetables (*n*=464). According to the Chinese dietary habits, rice and its products (*n* = 222) and wheat and its products (*n* = 47) are the two main groups in the cereals and potatoes category. For comparison, fish, shrimp, shellfish, eggs, livestock meat, and poultry were divided into three groups: fish, shrimp, and shellfish (*n* = 69), eggs (*n =* 117), and livestock meat and poultry (*n* = 342) for comparison. Among them, livestock meat and poultry were further divided into two groups: less than 100% can be eaten (*n* = 118) and 100% can be eaten (*n* = 224). The fruits were divided into two groups: fruit cut into pieces (*n* = 124) and whole fruit (*n =* 162). Vegetables were divided into root and stem vegetables (*n* = 75), melon and solanaceous vegetables (*n* = 129), mushroom and algae vegetables (*n* = 73), and leafy, flower, and sprout vegetables (*n* = 187).

As shown in Table 1, except for rice and its products, fish, shrimp, and shellfish, and livestock meat and poultry, where less than 100% can be eaten, the estimated d values of all food groups from the recall method were <0, indicating that the pregnant women underestimated the food weight. When using the WAIDA method, the estimated d values, except for fruits, were smaller and some food groups had positive values, indicating that the rectification of food weight using the WAIDA method was directional. The absolute value was further taken, and it was found that the means and SDs of D and D % obtained with the WAIDA method were lower than those obtained from the recall method, and the differences between the two methods were significant (*P* < 0.05), indicating that, compared with the weighing method, the variation range of food weight estimated by the WAIDA method was smaller and more stable than that estimated by the recall method.

Correlation analysis was subsequently performed. As shown in Table 2, the Pearson correlation coefficients between the food actual weight and the food weight estimated by the recall method ranged from −0.019 for livestock meat and poultry where less than 100% can be eaten, to 0.794 for wheat and its products, with an average of 0.46, and all correlations except for livestock meat and poultry where less than 100% can be eaten were statistically significant (*P* < 0.05). While the Pearson correlation coefficients between the food actual weight and the food weight estimated by the WAIDA method ranged from 0.332 for livestock meat and poultry where less than 100% can be eaten, to 0.973 for wheat and its products, with an average of 0.79, and all correlations were statistically significant (*P* < 0.001).

### 3.2. Comparison and Correlation Analysis of Energy and Nutrient Intakes Derived from the Recall or WAIDA Method with the Weighing Method

Based on the nutritional needs of women during pregnancy, energy, carbohydrate, protein, fat, total fatty acids, saturated fatty acids (SFA), monounsaturated fatty acids (MUFA), polyunsaturated fatty acids (PUFA), and representative vitamins (vitamin A, vitamin E, vitamin C, folic acid, vitamin B_6_, and vitamin B_12_) and minerals (calcium, magnesium, iron, zinc) were selected for further analysis. This part of the data was based on meals, including the 251 meals.

As shown in Table 3, except for vitamin B_12_, the estimated d values of energy and nutrients from the recall method were <0, indicating that the pregnant women underestimated the food weight, resulting in an underestimation of energy and nutrients. When using the WAIDA method, the estimated d values were small, except those for vitamin B_12_, and some nutrients had positive values, indicating that the rectification of energy and nutrients using the WAIDA method was directional, similar to the food weight method. The absolute value was further evaluated, and it was found that the means and SDs of D and D % obtained with the WAIDA method were lower than those obtained from the recall method, and the differences between the two methods were significant (*P* < 0.01), indicating that, compared with the weighing method, the variation range of energy and nutrients estimated by WAIDA method was smaller and more stable than that estimated by the recall method.

Correlation analysis was also performed. As shown in Table 4, the Pearson correlation coefficients between the energy and nutrient intakes estimated by the weighing method and those estimated by the recall method ranged from 0.480 for protein to 0.887 for vitamin B_6_, with an average of 0.68, and all correlations were statistically significant (*P* < 0.001). While the Pearson correlation coefficients of the energy and nutrient intakes estimated by the weighing method and those estimated by the WAIDA method ranged from 0.752 for zinc to 0.970 for vitamin B_12_, with an average of 0.871, and all correlations were statistically significant (*P* < 0.001).

### 3.3. Bland-Altman Analysis of Energy and Nutrient Intakes from Those Estimated by the Weighing Method and the Recall or WAIDA Method

**Figure 3:**
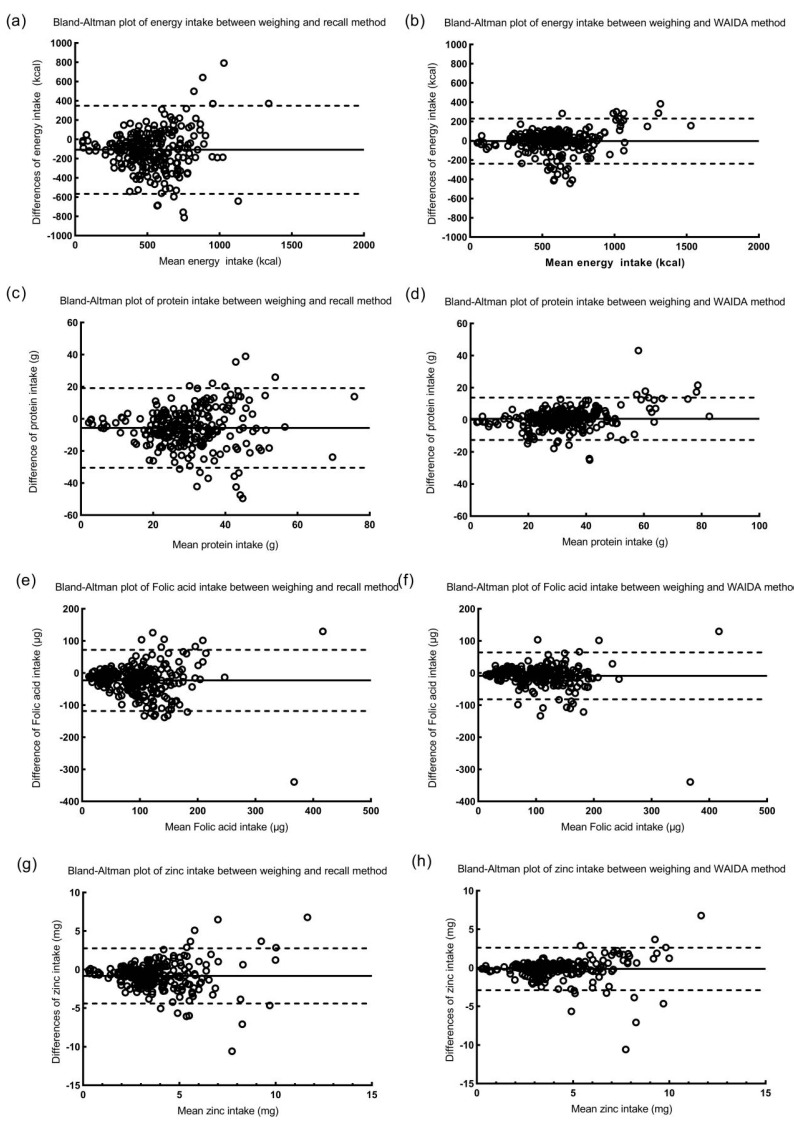
Bland-Altman plots showing the proportional biases of (**a**,**b**) energy, (**c**,**d**) protein, (**e**,**f**) folic acid, and (**g**,**h**) zinc intakes between the weighing method and recall or WAIDA method for 251 meals. The solid lines represent mean difference and the dashed lines represent the 95% limits of agreement (standard deviation 1.96).

To assess the agreement between the energy and nutrients estimated by the weighing method and those estimated by the recall or WAIDA method, a Bland-Altman analysis was performed. The mean difference and 95% LOAs for energy and nutrients are shown in Table 5. Overall, the mean differences of the energy and nutrients estimated from the recall method were further away from zero relative to those of the weighing method compared to the WAIDA method and with numerically wider 95% confidence intervals (CIs). The spans between the lower and upper LOAs of the energy and nutrients obtained by the recall method were broader than those obtained by the WAIDA method. Furthermore, Bland-Altman plots were used to show the relationship between the mean and the difference in the daily intake of energy and nutrients obtained from both the weighing method and the recall or WAIDA method. The x-axis represented the mean total intake of energy and nutrients from both the weighing method and the recall or WAIDA method, whereas the y-axis represented the difference in energy and nutrient intake between the two methods. A good agreement was defined as a situation in which no more than 10% of the points exceeded the 95% LOAs, and the points were close to the middle horizontal line. Although the spans between the upper and lower LOAs of the energy and nutrients obtained by the WAIDA method were generally narrower than those obtained by the recall method, the majority of the data points obtained by the WAIDA method lay between the LOAs, close to the middle horizontal line. The Pearson correlation coefficients between the nutrient intake estimated by the weighting method and those estimated by the recall method showed that protein was the lowest among the three macronutrients, folic acid was the lowest among vitamins, and zinc was the lowest among minerals. Therefore, the results for energy values and representative nutrients (protein, folic acid, and zinc) are shown in Figure 3.

## 4. Discussion

The present study established a novel WeChat Applet, called the WAIDA, to assess the diet of pregnant women in China. A total of 251 lunch meals of pregnant women in the second trimester were investigated to evaluate the relative validity of the WAIDA by analyzing the difference between the WAIDA and recall method when compared with the weighing method. The data analysis of food weight, energy, and nutrients showed that compared with the recall method, the data estimated by the WAIDA method showed less deviation and were closer to the actual values calculated using the weighing method. Therefore, it is feasible to evaluate the dietary quality, energy, and nutrient intake of pregnant women in order to provide a tool for antenatal care in China.

When developing a new dietary assessment, it is important to determine whether the estimation of food quantity deviates from the actual. Although the food validated in the present study was not representative of all food in China, the food commonly consumed by Chinese people was selected for this study. According to the Chinese Balanced Dietary Pagoda for pregnant women [22] and Chinese dietary habits, these foods could be divided into different categories and groups for comparative analysis. Our results showed that there was less deviation in the estimation of food quantity using the WAIDA method than the recall method, which is the goal we want to achieve. However, similar to previous image-based dietary assessments [26,27,28], the amount of food obtained by the WAIDA still showed a disparity with the actual food weight. This may be due to the relative ability of dietary assessors to identify and quantify food in images [10,19,29]. For example, indefinite-configuration foods such as dumplings, wonton, porridge, and soup, will make the estimation process cumbersome and complex, thus affecting the accuracy of the data. In further correlation analysis, one of the findings that attracted our attention is that the Pearson correlation coefficients between the actual food weight and the food weight estimated by the recall or WAIDA method were small for less than 100% can be eaten livestock and poultry food. As mentioned in the previous study, in the general population, there is a lack of relationship between the visual impression of the appearance of even 100% edible food and the weight of the corresponding food [30]. Therefore, in the recall method, the participants had no ability to accurately recall and estimate the weight of food with inedible parts, such as chicken legs, ribs, and fish, which led to a large error between the recall value and the real value. In the WAIDA method, the dietary assessors estimated the edible proportion of food based on the food atlas, in which the percentage of inedible food was fixed. However, the edible proportions of different parts of the food were different; using the average proportion data instead of the actual data may have a certain error.

We further calculated the energy and some important nutrient requirements for pregnant women. The d values of energy and most nutrients estimated by the recall method were less than 0. Combined with the results of food quantity, it was suggested that pregnant women underestimated the food weight, resulting in an underestimation of energy and nutrients. Our results showed that, compared with the recall method, there was less deviation in the estimation of energy and nutrients using the WAIDA method. The significant differences in energy and nutrient intake also reflected the accuracy of the two methods in terms of the variability between individual food consumption. In addition to significant differences, the correlation coefficients of energy and nutrients obtained by the WAIDA method and weighing method were also high. Taking energy as an example, the correlation coefficient between the values estimated by the WAIDA and weighing methods was 0.865, which is much higher than that estimated by the recall and weighing methods (0.509). The correlation coefficient of energy obtained by some previous image-based dietary assessment methods was lower than that of our research results. For example, Wang et al. reported the related research on 20 young women, with an energy correlation coefficient of 0.79 [31]. In the subsequent study, Wang et al. studied 28 young women in June and December, and the correlation coefficients of energy were 0.58 and 0.60, respectively [32]. These inconsistent results may be related to the research design, sample size, study population, dietary assessment methods, etc. However, high correlation does not necessarily indicate excellent consistency between methods; therefore, we used the Bland–Altman analysis to accurately evaluate the consistency of methods. The results were similar to those of previous image-based dietary assessment studies [33,34]. The Bland–Altman analysis showed that there was good consistency between the WAIDA method and the weighing method. Although plots for energy and each nutrient showed a few outliers, the majority of the measurements were scattered along the mean difference line. Comparing the mean difference and 95% LOAs of the difference between the recall method and the WAIDA method, the values of the WAIDA method were far lower than those of the recall method; in other words, the data estimated by the WAIDA method were less biased and closer to the true values.

Our study had several advantages. First, we focused on the evaluation of food rather than drinks. The main reason is that homemade drinks (boiled water, fruit juice, coffee, etc.) are usually poured into bottles or cups, and their quantity can be estimated according to measuring tools or standard tableware; Prepackaged drinks (milk, carbonated drinks, sugary drinks, etc.) purchased from supermarkets usually have a net content. It is much easier to estimate the intake of drinks than food. Second, in order to ensure the progress of the study, pregnant women in their second trimester were invited to participate in the study. The main reason is that in the first trimester of pregnancy, pregnant women will experience early pregnancy reactions with nausea, vomiting, loss of appetite and other symptoms. Some pregnant women need to stay in bed to protect their fetus. While in the third trimester of pregnancy, with the growth of the fetus, they will become more and more bulky, it becomes particularly difficult to do anything, and they face the risk of childbirth. Third, the weighing data were obtained by the researchers rather than by the participants themselves, which reduced the technical bias, improved the accuracy of the weighing data, and avoided the impact of the weighing records on the data obtained by the recall method. Fourth, unlike the previous image-based dietary assessments using images taken only at the front 45°, in our WAIDA method, the food was photographed from three angles: directly above, 45° angle in front and 45° angle behind, which was helpful for the assessors to make a comprehensive and three-dimensional evaluation of food, and reduce the errors in food type identification and food quantity estimation [26,29,35]. Fifth, we developed a food atlas for the WAIDA method based on Chinese cooking and processing methods. Each food was photographed from three different angles, and each picture showed four to six different food parts. This could improve the accuracy of food quantity estimations. Sixth, the WAIDA method has the same advantages as smartphone-based image dietary records; for example, it can reduce the burden on doctors and does not need pregnant women to estimate the portion size, which can improve the satisfaction of both doctors and pregnant women [7,13]. Unlike weighing records, diets do not require food to be weighed, so this method works well for pregnant women who often eat out of home. It can also avoid recall bias. Finally, it is necessary to evaluate the diet quality of pregnant women during the whole pregnancy. The WeChat software is widely used by young and middle-aged people in China. The direct transmission of images through the WeChat applet further simplifies image-based dietary assessment and can improve the compliance of pregnant women to take images of their diet over a long period of pregnancy and ensure the validity of these data [27,31,36].

In contrast, our study had several limitations. First, our research participants were pregnant women who often eat many small meals throughout the day. It must be better to investigate a whole day’s diet in the validation of WAIDA, but it’s too difficult to operate. In fact, the breakfast of Chinese people is relatively simple, and the ingredients for lunch and supper are sumptuous. As pregnant women generally go for antenatal care in the morning, lunch will be easier to operate in the hospital. Therefore, this study only investigated the relative validity of lunch meals, which does not represent the diet of a day. Second, although we conducted technical skill training for all the researchers, we did not check for inter- and intra- researcher variability. Because if a researcher is allowed to complete the study alone or repeat some steps involved in this study, there are many difficulties in the actual operation process, which can be realized only if the whole study is redone. For example, the food weighing of lunch preparation was completed by three researchers in charge of this work together. During the dietary recall, if the same researcher asks pregnant women twice or different researchers ask pregnant women respectively, pregnant women will not cooperate well. Therefore, we carried out quality control. After the interview, the researchers checked for any unclear information that the pregnant women had returned and resolved any issue. In the process of identifying and quantifying the food in each image, different dietary assessors may have different results when evaluating the same image. Therefore, we have conducted periodic assessment on dietary assessors to keep the error between their estimated weight and the real weight of food within 10%. Finally, studies conducted to validate dietary assessments require dietary nutritional biomarkers that reflect a wide selection of food items. Our study is just an initial research, and thus biomarkers were not involved.

## 5. Conclusions

This validation study demonstrated that the WAIDA method is a simple and effective method for dietary evaluation. Future studies should explore the validity of the WAIDA in a larger, more representative sample and employ nutritional biomarkers of diet to reflect the usual intake. This would confirm its value as a tool to monitor the dietary quality, energy, and nutrient intake of pregnant women in antenatal care in China. As we are living more and more in a digital world, in addition to WeChat software, we can also use other ways in the future, such as YouTube, so that this method of dietary assessment will be accepted by people all over the world.

## Figures and Tables

**Table 1:** Comparison of the food weight estimated by the recall or WAIDA method with the actual food weight (Mean ± SD).

Food Groups	*n*	Methods	d (g)	D (g)	d% (%)	D% (%)
**Cereals and potatoes**	269	Recall	−19.95 ± 40.82	33.99 ± 30.11	18.14 ± 42.77	37.58 ± 27.24
		WAIDA	−6.80 ± 15.56 *	13.33 ± 10.50 *	−8.42 ± 21.19 **	16.54 ± 15.67 **
Rice and its products	222	Recall	23.42 ± 41.56	37.24 ± 29.76	23.57 ± 38.90	37.94 ± 25.00
		WAIDA	−9.151 ± 14.98 **	14.25 ± 10.22 **	−9.84 ± 17.04 **	15.61 ± 11.94 **
Wheat and its products	47	Recall	−6.86 ± 34.66	20.50 ± 28.64	2.33 ± 47.14	34.80 ± 31.48
		WAIDA	−2.43 ± 14.58	9.29 ± 11.42 **	5.07 ± 33.51	19.94 ± 27.26 **
**Fish, shrimp, shellfish, eggs, livestock meat, and poultry**	528	Recall	−4.75 ± 42.88	30.57 ± 30.42	−1.16 ± 76.83	53.39 ± 55.21
		WAIDA	1.42 ± 20.09 **	15.28 ± 13.10 **	4.64 ± 38.08	28.39 ± 25.78 **
Fish, shrimp, and shellfish	69	Recall	17.74 ± 55.74	42.18 ± 40.26	50.43 ± 104.11	80.13 ± 83.10
		WAIDA	10.63 ± 17.97	15.04 ± 14.42 **	22.50 ± 35.89 *	29.78 ± 30.03 **
Eggs	117	Recall	−15.34 ± 27.96	24.12 ± 20.78	−18.73 ± 61.87	42.80 ± 48.30
		WAIDA	−6.21 ± 18.51 **	15.35 ± 12.00 **	−5.43 ± 37.71 *	27.73 ± 26.01 **
Livestock meat and poultry	342	Recall	−5.67 ± 42.60	30.43 ± 30.31	−5.56 ± 70.68	51.62 ± 48.52
		WAIDA	2.17 ± 21.13 **	15.31 ± 13.22 **	4.48 ± 37.44 **	28.34 ± 24.82 **
Less than 100% can be eaten	118	Recall	14.10 ± 53.22	38.56 ± 39.16	29.29 ± 88.74	61.19 ± 70.45
		WAIDA	9.35 ± 22.01	19.12 ± 14.28 **	18.53 ± 37.47	31.58 ± 27.29 **
100% can be eaten	224	Recall	−16.09 ± 31.20	26.14 ± 23.38	−23.92 ± 50.27	46.58 ± 30.36
		WAIDA	−1.62 ± 18.00 **	13.31 ± 12.19 **	−2.92 ± 35.32 **	26.63 ± 23.31 **
**Fruits**	286	Recall	−6.70 ± 46.27	30.41 ± 35.46	−3.03 ± 57.04	40.57 ± 40.13
		WAIDA	−10.10 ± 23.10	18.32 ± 17.29 **	−9.93 ± 26.88 *	22.72 ± 17.41 **
Fruit cut into pieces	124	Recall	−13.51 ± 57.75	37.88 ± 45.52	−9.93 ± 65.33	47.64 ± 45.60
		WAIDA	−8.80 ± 27.08	20.20 ± 20.01 **	−5.58 ± 30.17	23.88 ± 19.15 **
Whole fruit	162	Recall	−1.48 ± 34.32	24.70 ± 23.80	2.25 ± 49.34	35.16 ± 34.58
		WAIDA	−11.09 ± 19.54 **	16.89 ± 14.79 **	−13.26 ± 23.62 **	21.84 ± 15.96 **
**Vegetables**	464	Recall	−11.71 ± 43.76	32.32 ± 31.71	−11.22 ± 60.22	49.10 ± 36.56
		WAIDA	−1.18 ± 25.51 **	17.82 ± 18.27 **	5.71 ± 42.60 **	30.13 ± 30.63 **
Root and stem vegetables	75	Recall	−5.69 ± 42.26	27.75 ± 32.22	−3.93 ± 52.44	42.01 ± 31.25
		WAIDA	5.44 ± 25.36 *	15.89 ± 20.43 **	10.27 ± 35.07 *	26.05 ± 25.47 **
Melon and solanaceous vegetables	129	Recall	−6.17 ± 48.90	34.93 ± 34.64	−4.85 ± 57.35	45.75 ± 34.70
		WAIDA	−1.81 ± 31.27	21.88 ± 22.34 **	12.97 ± 49.82 **	33.82 ± 38.72 **
Mushrooms and algae vegetables	73	Recall	−8.10 ± 19.64	15.09 ± 14.88	−7.15 ± 74.21	55.48 ± 49.38
		WAIDA	0.19 ± 12.50 **	9.16 ± 8.44 **	5.57 ± 53.33	36.30 ± 39.26 **
Leafy, flower, and sprout vegetables	187	Recall	−18.78 ± 46.32	38.68 ± 31.56	−19.06 ± 59.09	51.67 ± 34.25
		WAIDA	−4.15 ± 24.19 **	18.96 ± 15.53 **	−1.87 ± 32.98 **	26.61 ± 19.46 **
**All Kinds of food**	1547	Recall	−9.87 ± 43.74	31.67 ± 31.73	−7.50 ± 63.64	46.94 ± 43.61
		WAIDA	−0.52 ± 22.34 **	16.25 ± 15.33 **	2.94 ± 35.82 **	25.75 ± 25.06 **

WAIDA: Wechat Applet for Image-based Dietary Assessment; d: relative difference; D: absolute difference; SD: standard deviation. d (g)= estimated weight (g) − actual weight (g); D (g)= |estimated weight (g) − actual weight (g)|. d% = [(estimated weight − actual weight) (g)/actual weight (g)] × 100; D% = [|estimated weight − actual weight| (g)/actual weight (g)] × 100. For each food, the differences in weight estimation with the recall and WAIDA method were compared using a paired *t*-test; * *P* < 0.05, ** *P* < 0.01.

**Table 2:** Pearson correlation coefficients between the actual food weight and the food weight estimated by the recall or WAIDA method.

Food Groups	*n*	Recall		WAIDA	
		*r*	*P*	*r*	*P*
**Cereals and potatoes**	269	0.545	<0.001	0.916	<0.001
Rice and its products	222	0.46	<0.001	0.871	<0.001
Wheat and its products	47	0.794	<0.001	0.973	<0.001
**Fish, shrimp, shellfish, eggs, livestock meat, and poultry**	528	0.359	<0.001	0.781	<0.001
Fish, shrimp, and shellfish	69	0.383	0.001	0.864	<0.001
Eggs	117	0.218	0.018	0.589	<0.001
Livestock meat and poultry	342	0.387	<0.001	0.804	<0.001
Less than 100% can be eaten	118	−0.019	0.836	0.332	<0.001
100% can be eaten	224	0.543	<0.001	0.869	<0.001
**Fruits**	286	0.37	<0.001	0.753	<0.001
Fruit cut into pieces	124	0.309	<0.001	0.739	<0.001
Whole fruit	162	0.479	<0.001	0.774	<0.001
**Vegetables**	464	0.635	<0.001	0.818	<0.001
Root and stem vegetables	75	0.626	<0.001	0.854	<0.001
Melon and solanaceous vegetables	129	0.745	<0.001	0.812	<0.001
Mushrooms and algae vegetables	73	0.534	<0.001	0.876	<0.001
Leafy, flower, and sprout vegetables	187	0.371	<0.001	0.709	<0.001
**All Kinds of food**	1547	0.520	<0.001	0.825	<0.001

WAIDA: Wechat Applet for Image-based Dietary Assessment.

**Table 3:** Comparison of energy and nutrient intakes derived from the recall or WAIDA method with the weighing method (*n* = 251 meals, Mean ± SD).

Food Groups	Methods	d	D	d% (%)	D% (%)
Energy (kcal)	Recall	−107.79 ± 233.32	186.42 ± 176.66	−16.41 ± 35.23	30.85 ± 23.57
	WAIDA	−3.28 ± 119.36 **	80.94 ± 87.64 **	0.41 ± 19.41 **	13.98 ± 13.43 **
Carbohydrate (g)	Recall	−10.17 ± 31.99	20.32 ± 26.70	−16.09 ± 41.53	32.22 ± 30.69
	WAIDA	−2.22 ± 14.89 **	9.23 ± 11.88 **	−1.28 ± 20.19 **	14.09 ± 14.49 **
Protein (g)	Recall	−5.66 ± 12.65	10.53 ± 8.99	−14.20 ± 36.45	31.70 ± 22.84
	WAIDA	0.66 ± 6.75 **	4.57 ± 5.00 **	1.41 ± 20.62 **	14.80 ± 14.40 **
Fat (g)	Recall	−5.08 ± 12.23	9.44 ± 9.28	−15.39 ± 46.82	38.19 ± 31.08
	WAIDA	0.31 ± 5.94 **	4.15 ± 4.26 **	1.25 ± 22.89 **	17.89 ± 14.30 **
Total fatty acid (g)	Recall	−5.24 ± 10.60	8.37 ± 8.33	−18.98 ± 45.78	38.70 ± 30.89
	WAIDA	−0.90 ± 6.67 **	4.41 ± 5.06 **	−3.12 ± 29.04 **	21.38 ± 19.86 **
SFA (g) ¹	Recall	−2.21 ± 4.39	3.52 ± 3.42	−19.07 ± 46.34	39.89 ± 31.52
	WAIDA	−0.38 ± 2.80 **	1.84 ± 2.14 **	−2.84 ± 29.80 **	21.46 ± 20.82 **
MUFA (g) ²	Recall	−2.39 ± 5.09	3.90 ± 4.04	−18.43 ± 48.39	40.35 ± 32.38
	WAIDA	−0.40 ± 3.20 **	2.10 ± 2.44 **	−2.64 ± 30.96 **	22.70 ± 21.17 **
PUFA (g) ³	Recall	−0.54 ± 1.06	0.85 ± 0.83	−18.48 ± 40.90	35.49 ± 27.41
	WAIDA	−0.11 ± 0.73 **	0.46 ± 0.57 **	−3.90 ± 26.64 **	20.08 ± 17.89 **
Vitamin A (μg RAE) ^4^	Recall	−42.22 ± 95.25	61.01 ± 84.41	−18.66 ± 37.43	34.58 ± 23.45
	WAIDA	−14.57 ± 73.67 **	37.64 ± 64.95 **	−4.00 ± 28.52 **	21.69 ± 18.90 **
Vitamin E (mg)	Recall	−0.55 ± 1.33	0.90 ± 1.12	−15.80 ± 36.42	32.30 ± 23.02
	WAIDA	−0.31 ± 1.18 **	0.54 ± 1.10 **	−5.43 ± 25.82 **	17.97 ± 19.30 **
Vitamin C (mg)	Recall	−5.61 ± 21.20	16.04 ± 14.93	−10.53 ± 45.48	36.66 ± 28.82
	WAIDA	−3.56 ± 15.22	9.82 ± 12.14 **	−4.65 ± 34.91 **	23.32 ± 26.36 **
Folic acid (μg)	Recall	−22.94 ± 48.54	37.46 ± 38.42	−17.70 ± 39.84	34.11 ± 27.09
	WAIDA	−8.83 ± 37.33 **	22.03 ± 31.38 **	−3.89 ± 28.76 **	20.05 ± 20.94 **
Vitamin B_6_ (mg)	Recall	−0.21 ± 0.41	0.32 ± 0.32	−18.86 ± 40.40	35.64 ± 26.72
	WAIDA	−0.06 ± 0.33 **	0.18 ± 0.28 **	−2.77 ± 27.75 **	19.33 ± 20.06 **
Vitamin B_12_ (μg)	Recall	0.12 ± 2.52	1.18 ± 2.23	−13.84 ± 57.24	41.96 ± 41.24
	WAIDA	0.25 ± 1.12	0.59 ± 0.98 **	2.60 ± 42.94 **	23.60 ± 35.93 **
Calcium (mg)	Recall	−24.27 ± 50.90	37.35 ± 42.20	−17.14 ± 33.67	30.89 ± 21.70
	WAIDA	−7.81 ± 43.16 **	23.49 ± 37.01 **	−4.24 ± 24.34 **	17.76 ± 17.13 **
Magnesium (mg)	Recall	−40.41 ± 151.45	58.28 ± 145.49	−15.41 ± 32.00	28.79 ± 20.74
	WAIDA	−1.32 ± 82.08 **	30.80 ± 76.07 **	−3.53 ± 20.48 **	15.18 ± 14.16 **
Iron (mg)	Recall	−6.79 ± 33.10	9.28 ± 32.47	−17.56 ± 36.00	31.88 ± 24.19
	WAIDA	0.62 ± 17.27 **	4.72 ± 16.62 **	−4.73 ± 23.38 **	16.21 ± 17.47 **
Zinc (mg)	Recall	−0.82 ± 1.83	1.43 ± 1.40	−16.44 ± 36.27	32.38 ± 23.11
	WAIDA	−0.14 ± 1.41 **	0.78 ± 1.18 **	−2.14 ± 23.40 **	16.81 ± 16.38 **

WAIDA: Wechat Applet for Image-based Dietary Assessment; d: relative difference; D: absolute difference; SD: standard deviation. d = energy and nutrient intakes derived from the recall or WAIDA method − energy and nutrient intakes derived from the weighing method; D = |energy and nutrient intakes derived from the recall or WAIDA method − energy and nutrient intakes derived from the weighing method|; d% = [(energy and nutrient intakes derived from the recall or WAIDA method − energy and nutrient intakes derived from the weighing method)/energy and nutrient intakes derived from the weighing method] × 100; D% = (|energy and nutrient intakes derived from the recall or WAIDA method − energy and nutrient intakes derived from the weighing method|/energy and nutrient intakes derived from the weighing method) × 100; ¹ SFA: saturated fatty acids; ² MUFA: monounsaturated fatty acids; ³ PUFA: polyunsaturated fatty acids; ^4^ RAE: retinol activity equivalent; For energy and each nutrient, the differences in estimation with the recall and WAIDA method were compared using a paired *t*-test; ** *P* < 0.01.

**Table 4:** Pearson correlation coefficients of energy and nutrient intakes from weighing method and those estimated by the recall or WAIDA method (*n* = 251 meals).

Energy and Nutrients	Recall		WAIDA	
	r	*P*	r	*P*
Energy (kcal)	0.509	<0.001	0.865	<0.001
Carbohydrate (g)	0.584	<0.001	0.860	<0.001
Protein (g)	0.48	<0.001	0.887	<0.001
Fat (g)	0.576	<0.001	0.917	<0.001
Total fatty acid (g)	0.593	<0.001	0.863	<0.001
SFA ¹ (g)	0.592	<0.001	0.854	<0.001
MUFA ² (g)	0.596	<0.001	0.866	<0.001
PUFA ³ (g)	0.605	<0.001	0.840	<0.001
Vitamin A (μg RAE ^4^)	0.815	<0.001	0.893	<0.001
Vitamin E (mg)	0.722	<0.001	0.788	<0.001
Vitamin C (mg)	0.676	<0.001	0.797	<0.001
Folic acid (μg)	0.656	<0.001	0.792	<0.001
Vitamin B_6_ (mg)	0.887	<0.001	0.928	<0.001
Vitamin B_12_ (μg)	0.865	<0.001	0.970	<0.001
Calcium (mg)	0.808	<0.001	0.866	<0.001
Magnesium (mg)	0.821	<0.001	0.963	<0.001
Iron (mg)	0.844	<0.001	0.969	<0.001
Zinc (mg)	0.555	<0.001	0.752	<0.001

WAIDA: Wechat Applet for Image-based Dietary Assessment; ^1^ SFA: saturated fatty acids; ^2^ MUFA: monounsaturated fatty acids; ^3^ PUFA: polyunsaturated fatty acids; ^4^ RAE: retinol activity equivalent.

**Table 5:** Bland-Altman analyses of energy and nutrient intakes estimated by the weighing method and those estimated by the recall or WAIDA method (*n* = 251 meals).

Energy and Nutrients	Weighing Method VS Recall Method			Weighing Method VS WAIDA Method		
	Mean Differences (95% Confidence Interval)	95%LOA ^1^		Mean Differences (95% Confidence Interval)	95%LOA	
		Lower Limit	Upper Limit		Lower Limit	Upper Limit
Energy (kcal)	−107.8 (−136.79; −78.78)	−565.1	349.5	−3.28 (−18.12; 11.56)	−237.2	230.7
Carbohydrate (g)	−10.17 (−14.15; −6.13)	−72.87	52.53	−2.22 (−4.07; −0.37)	−31.41	26.96
Protein (g)	−5.66 (−5.47; −4.09)	−30.45	19.12	0.66 (−0.18; 1.49)	−12.57	13.88
Fat (g)	−5.08 (−6.60; −3.56)	−29.06	18.89	0.31 (−0.43; 1.05)	−11.33	11.95
Total fatty acid (g)	−5.24 (−6.56; −3.92)	−26.01	15.53	−0.90 (−1.73; −0.07)	−13.97	12.17
SFA ^2^ (g)	−2.21 (−2.75; −1.66)	−10.80	6.39	−0.38 (−0.73; −0.03)	−5.86	5.10
MUFA ^3^ (g)	−2.39 (−3.02; −1.75)	−12.36	7.59	−0.40 (−0.79; 0.00)	−6.66	5.87
PUFA ^4^ (g)	−0.54 (−0.67; −0.41)	−2.62	1.54	−0.11 (−0.20; −0.02)	−1.54	1.31
Vitamin A (μg RAE ^5^)	−42.20 (−54.06; −30.38)	−228.9	144.5	−14.57 (−23.73; −5.41)	−159.0	129.8
Vitamin E (mg)	−0.55 (−0.71; −0.38)	−3.15	2.05	−0.31 (−0.46; −0.16)	−2.63	2.01
Vitamin C (mg)	−5.61 (−8.25; −2.98)	−47.16	35.95	−3.56 (−5.45; −1.67)	−33.38	26.27
Folic acid (μg)	−22.94 (−28.98; −16.91)	−118.1	72.20	−8.83 (−13.47; −4.19)	−82.00	64.34
Vitamin B_6_ (mg)	−0.21 (−0.26; −0.16)	−1.00	0.59	−0.06 (−0.10; −0.02)	−0.71	0.58
Vitamin B_12_ (μg)	0.12 (−0.19; 0.44)	−4.81	5.06	0.25 (0.11;0.39)	−1.93	2.44
Calcium (mg)	−24.26 (−30.59; −17.94)	−124.0	75.49	−7.81 (−13.17; −2.44)	−92.4	76.78
Magnesium (mg)	−40.41 (−59.24; −21.58)	−337.2	256.4	−1.32 (−11.53; 8.88)	−162.2	159.5
Iron (mg)	−6.79 (−10.91; −2.68)	−71.67	58.09	0.62 (−1.53; 2.77)	−33.23	34.47
Zinc (mg)	−0.82 (−1.05; −0.60)	−4.41	2.76	−0.14 (−0.32; 0.03)	−2.90	2.62

WAIDA: Wechat Applet for Image-based Dietary Assessment; ¹ LOA: limits of agreement; SD: standard deviation; ² SFA: saturated fatty acids; ^3^ MUFA: monounsaturated fatty acids; ^4^ PUFA: polyunsaturated fatty acids; ^5^ RAE: retinol activity equivalent.

## Data Availability

The data presented in this study are available upon request from the corresponding author.
